# The Anatomical and Physiological Basis of Clinical Tests of Otolith Function. A Tribute to Yoshio Uchino

**DOI:** 10.3389/fneur.2020.566895

**Published:** 2020-10-20

**Authors:** Ian S. Curthoys

**Affiliations:** Vestibular Research Laboratory, School of Psychology, The University of Sydney, Sydney, NSW, Australia

**Keywords:** vestibular, otolith, utricular, saccular, vemp

## Abstract

Otolithic receptors are stimulated by gravitoinertial force (GIF) acting on the otoconia resulting in deflections of the hair bundles of otolithic receptor hair cells. The GIF is the sum of gravitational force and the inertial force due to linear acceleration. The usual clinical and experimental tests of otolith function have used GIFs (roll tilts re gravity or linear accelerations) as test stimuli. However, the opposite polarization of receptors across each otolithic macula is puzzling since a GIF directed across the otolith macula will excite receptors on one side of the line of polarity reversal (LPR at the striola) and simultaneously act to silence receptors on the opposite side of the LPR. It would seem the two neural signals from the one otolith macula should cancel. In fact, Uchino showed that instead of canceling, the simultaneous stimulation of the oppositely polarized hair cells enhances the otolithic response to GIF—both in the saccular macula and the utricular macula. For the utricular system there is also commissural inhibitory interaction between the utricular maculae in each ear. The results are that the one GIF stimulus will cause direct excitation of utricular receptors in the activated sector in one ear as well as indirect excitation resulting from the disfacilitation of utricular receptors in the corresponding sector on the opposite labyrinth. There are effectively two complementary parallel otolithic afferent systems—the sustained system concerned with signaling low frequency GIF stimuli such as roll head tilts and the transient system which is activated by sound and vibration. Clinical tests of the sustained otolith system—such as ocular counterrolling to roll-tilt or tests using linear translation—do not show unilateral otolithic loss reliably, whereas tests of transient otolith function [vestibular evoked myogenic potentials (VEMPs) to brief sound and vibration stimuli] do show unilateral otolithic loss. The opposing sectors of the maculae also explain the results of galvanic vestibular stimulation (GVS) where bilateral mastoid galvanic stimulation causes ocular torsion position similar to the otolithic response to GIF. However, GVS stimulates canal afferents as well as otolithic afferents so the eye movement response is complex.

## Introduction

The canals and the otolithic sensory regions of the inner ear function as an integrated system—in response to head movements, otolith signals interact with canal signals to generate appropriate sensations, eye movements, and postural responses (1). Loss of otolith function disrupts that neural interaction and causes patient reports of disorientation as well as postural unsteadiness (2). Many patients arrive at clinics complaining of dizziness, vertigo, postural unsteadiness but tests in these patients may show all semicircular canals have normal function (2).

In parallel with the aim of clinical testing of the semicircular canals, clinical tests of otolith function seek to identify the level of otolith function in each ear and whether there is unilateral loss of otolith function. However, the structure of the otoliths is unusual and, as we show below, tests which *prima facie* appear that they should be indicators of the level of otolith function in each ear do not provide clinically useful data about asymmetry of otolith function. In particular, the opposite polarization of receptors across each otolithic macula is puzzling since a gravitoinertial force (GIF) stimulus directed across the otolith macula will excite receptors on one side of the line of polarity reversal (LPR at the striola) and simultaneously act to silence receptors on the opposite side of the LPR. It would seem the two neural signals from the one otolith macula should cancel. In fact, Uchino's detailed physiology in the VN show exactly the opposite!—that instead of canceling, the simultaneous stimulation of the oppositely polarized hair cells enhances the otolithic response to GIF—both in the saccular macula and the utricular macula.

In the labyrinth of each ear the otolithic receptors are laid out on two sheets of cells called maculae—the utricular macula and the saccular macula—and the receptors and afferents within each macula form two complementary otolithic systems—the sustained system concerned with signaling low frequency GIFs and the transient system which is activated by high frequency stimuli such as sounds and vibration (1). Tests of the sustained otolith system do not show unilateral loss reliably, whereas tests of transient (dynamic) otolith function do show unilateral otolithic loss.

**Figure 1:**
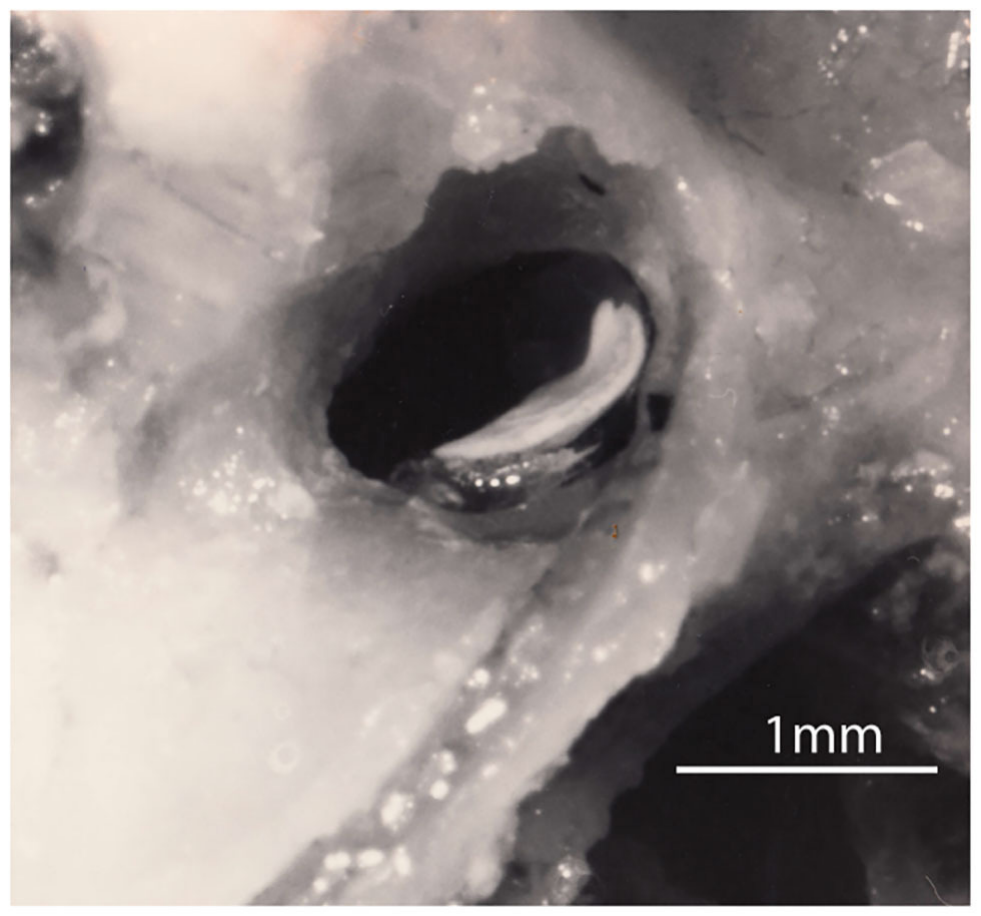
A lateral view of the right utricular macula in a guinea pig. The white layer is the otoconia adhering to the upper surface of the gelatinous otoconial membrane (OM) on the macula. The upturn is at the rostral end of the macula where it is attached to bone and where the afferent neurons leave. The rest of the macula (the flat plate) rests on a membrane stretched across the labyrinth (the *membrana limitans*) so that most of the utricular macula effectively floats on fluid (3–5). Figure reproduced with permission of the Aerospace Medical Association from Curthoys (3).

**Figure 2:**
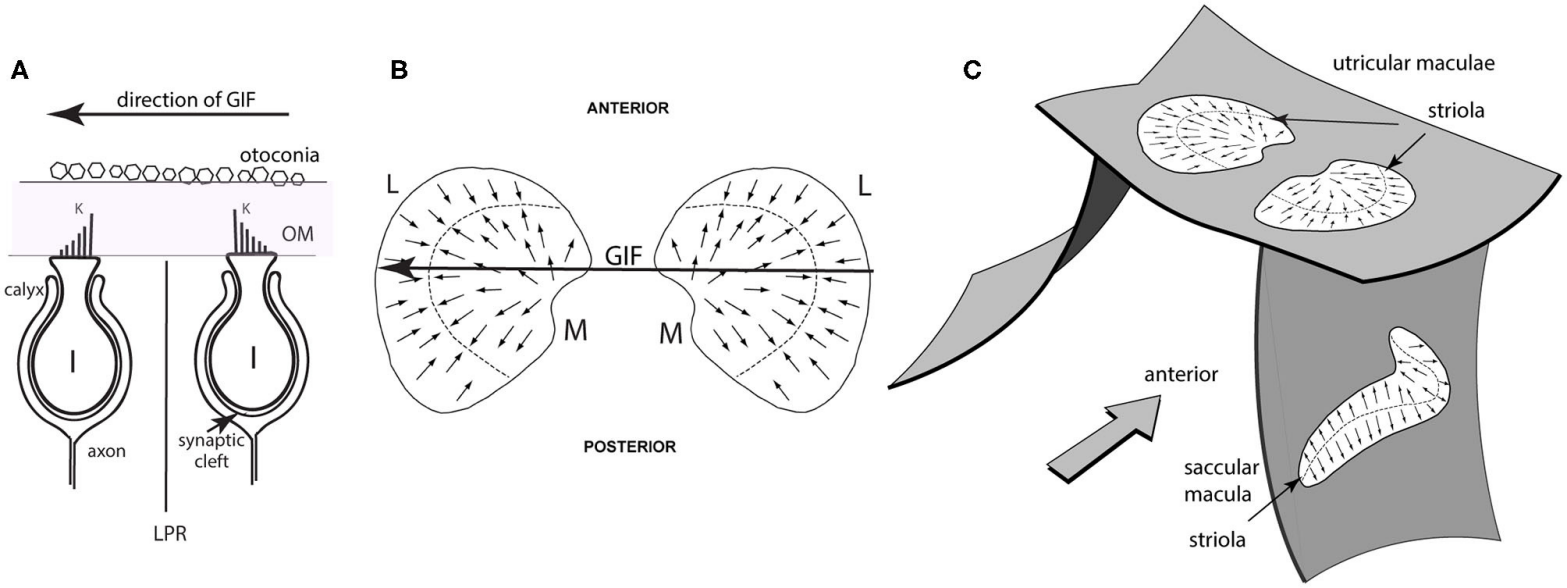
Simplified schematic diagrams of otolithic type I receptors, the line of polarity reversal and the spatial organization of the utricular and saccular maculae. **(A)** Showing two type I receptors on either side of the line of polarity reversal (LPR). The primary afferent neuron forms a calyx ending which envelops the whole amphora-shaped receptor cell body. Other receptors are barrel shaped and are called type II receptors. The kinocilium is the longest cilium and is identified by the letter K in this schematic. A gravitoinertial force directed from right to left (such as a roll tilt of the head left ear down) will displace the otoconia above the macula which will deflect the hair bundles of the receptors and so facilitate the type I receptor on the right because the cilia of that receptor will be deflected toward the kinocilium, but simultaneously disfacilitate the receptor on the left whose cilia will be deflected away from the kinocilium. **(B)** Schematic representations of the view looking straight down on the two utricular maculae during a laterally directed GIF to the left (shown by the large arrow). The small arrows represent the preferred directions of receptors on the maculae and the systematic change in preferred direction and the opposite polarization on either side of the line of polarity reversal (LPR—dashed line) are shown. The two utricular maculae are mirror images of one another. The medial sectors are identified by the letter M and the lateral sectors by the letter L. The GIF (large arrow) simultaneously facilitates left medial and disfacilitates left lateral receptors. **(C)** Showing the approximate spatial configuration of the utricular and saccular maculae in the head. The band of receptors adjacent to the line of polarity reversal is called the striola. In the utricular macula the receptors point toward the LPR, whereas in the saccular macula the receptors point away from the LPR [from Curthoys (6), Copyright © 2020 Karger Publishers, Basel, Switzerland].

A good example of a test using the sustained system is the response to maintained head tilt. Figure 1 shows a side-on view of the utricular macula of a guinea pig—the white layer being the otoconia adhering to the upper surface of the otolithic membrane. The human utricular macula is similar. During a roll head tilt, gravity displaces the crystals (the otoconia) and so stimulates the otolith utricular receptors (Figure 2A) and causes both eyes to roll around the line of sight and to maintain a rolled position during the maintained head tilt. A roll head tilt, left ear down, causes both eyes to roll so the upper pole of both eyes is rolled in the orbit by a few degrees to the right. This response is termed ocular torsion or ocular counterrolling (OCR). It has been presumed that loss of the otoliths in one labyrinth should result in asymmetrical OCR for the two directions of lateral head tilt, just as unilateral loss of the semicircular canals results in asymmetrical horizontal vestibulo-ocular responses. At the acute stage roll head tilts to the affected side do show reduced OCR, but that is not the case in patients with long term unilateral loss—there is no systematic asymmetry in OCR responses for roll-tilts to the left or right (7). However, tests of the transient otolith system, do show unilateral otolithic loss acutely and chronically. In this review we examine the peripheral anatomy and physiology of the otoliths underlying these very different and puzzling outcomes.

### Summary of Otolith Anatomy and Physiology

#### Vestibular Receptor Hair Cells

In the human there are around 33,000 receptors in each utricular macula [synapsing on around 6,000 afferents (8)] and 18,000 receptors in each saccular macula (9) [synapsing on around 4,000 saccular afferents (8)]. Projecting from each otolithic receptor cell are hair-like cilia and deflections of these hair bundles stimulate the receptor. The hair bundles project into the gelatinous otoconial membrane (OM), the upper surface of which is covered by otoconia (Figures 1, 2A). Each receptor has one distinct cilium (the kinocilium, K) which serves as a unique feature which identifies the preferred direction of stimulation of that receptor—its “morphological polarization” or directional preference (10). Intracellular recording from isolated receptors has shown that for all receptors, deflections of the receptor hair bundle toward the kinocilium are facilitatory (excitatory), deflections away from the kinocilium are disfacilitatory (11–13) (Figure 2A). There is a systematic change in the directional preference of the individual receptors around the utricular macula (9). This structural organization is shown schematically in Figures 2B,C as small arrows on the surface of the maculae, representing the different preferred directions of receptors all over the macula. This spatial ordering of the directional preference of receptors in the otolithic maculae (Figure 2C) contrasts with the uniform directional preference of all receptors on each semicircular canal crista (9). Each otolithic maculae is divided into two sectors in which the hair cells have exactly opposite directional preferences (9) (Figures 2B,C).

The line dividing the two sectors is called the line of polarity reversal (LPR) and the thin band of receptors on either side of the LPR is called the striola.

Gravity is usually the stimulus generating hair bundle deflection. A gravitoinertial force in one direction displaces the dense crystals of the otoconia of the otolith organs, and so the hair bundles of the otolithic receptor hair cells, embedded in the otoconial membrane, tuned to that direction are deflected and activated. Recently it has been shown that sound and vibration are very effective stimuli for one class of otolithic receptors and afferents—those with irregular resting discharge originating from receptors at the striola (14). Other stimuli [small electric currents called galvanic vestibular stimulation (GVS) delivered by surface electrodes on the mastoids] activate all vestibular receptors and afferent neurons on the side of the cathode electrode and inhibit afferents on the side of the anode electrode (15–17). Each one of these stimuli has been used in possible clinical tests of otolithic function and they are discussed below after considering the anatomy and physiology of the otoliths.

**Figure 3:**
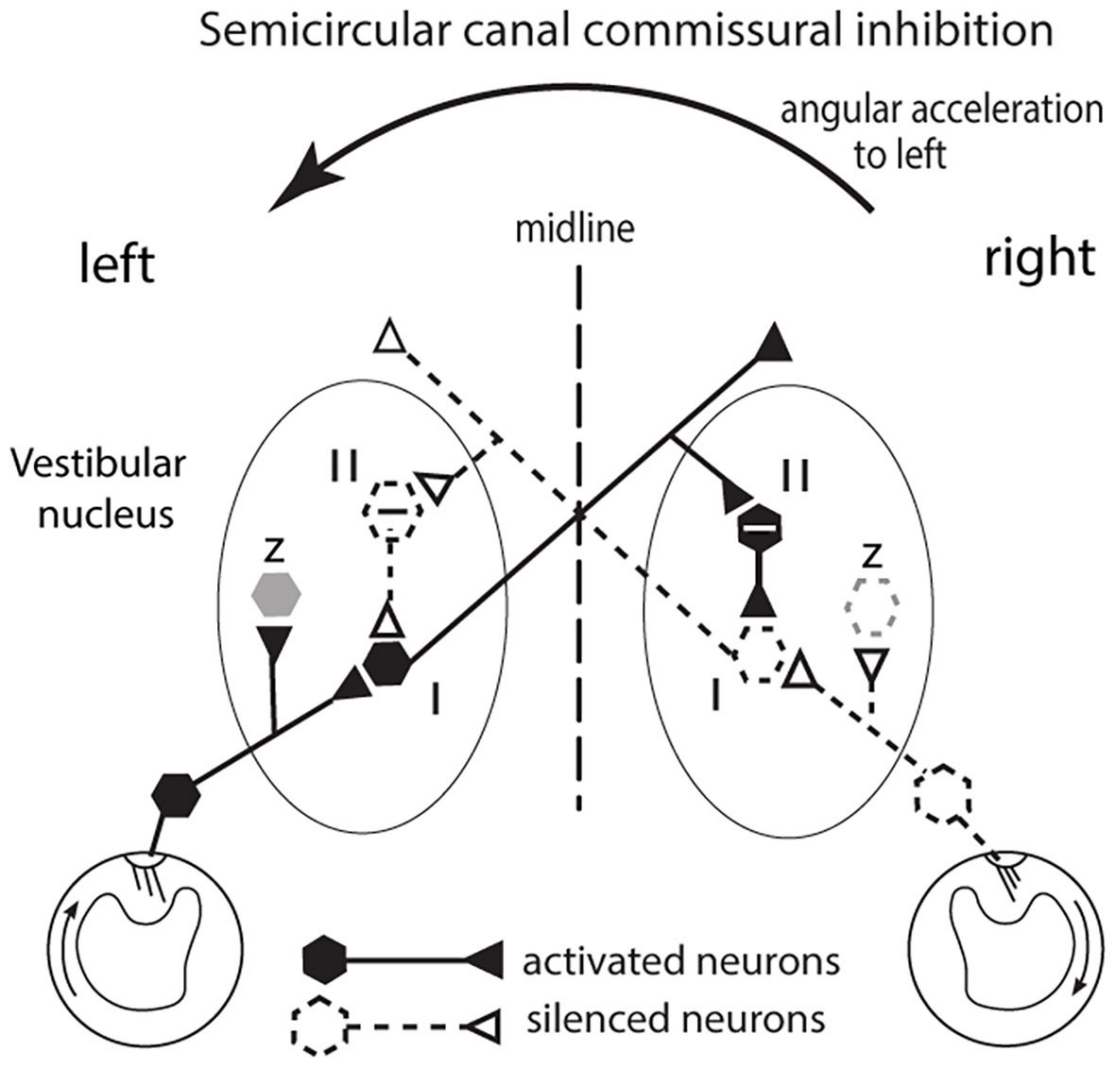
The commissural inhibitory interaction of the semicircular canals to angular acceleration during a leftward head turn. The figure represents a view looking down on the horizontal canals and the vestibular nuclei of the brainstem. The primary afferents from the horizontal canal on the side to which the head is turned (left in this example) are facilitated (activated) and in turn they activate VN type I neurons (excitatory neurons receiving afferent input). Simultaneously the corresponding primary afferents on the opposite (right) side are silenced (disfacilitated) because the fluid flow deflects the cilia away from the kinocilium and so the VN neurons they serve are disfacilitated [Conventions: solid lines and hexagons—neurons which are activated; dashed lines and hexagons—neurons whose activity (firing rate) is reduced or silenced; hexagons containing a—sign are inhibitory neurons]. The result is that the angular acceleration causes an imbalance in the neural activity of type I neurons in the two VN. That imbalance is enhanced by an axon branch from the activated left type I projecting to the right side, activating an inhibitory neuron (type II) and the increased inhibition it exerts on its target type I neuron further silences the type I neuron on the right side. In this way the imbalance occurring at the periphery is further enhanced. In turn the silencing of those right VN type I neurons acts to reduce inhibition on the left VN type I neuron and so acts to increase its firing and so further enhance the difference in neural activity between the two VN. That release from inhibition is called disinhibition. Note that this “closed loop” depicts the activity of only a small group of canal neurons in the VN: other VN neurons (shown as z in the figure and shown as light gray) are outside this closed loop. These data are based on results by Shimazu and Precht (18); Markham et al. (19).

The receptor organization of the otolithic maculae means that in response to the one GIF stimulus, some otolithic receptors and afferents have an increased activation (facilitation) whereas others in the same macula have decreased activation (disfacilitation) to exactly the same stimulus (Figure 2B). It seems that these two opposite responses should cancel. In fact the opposite is true—Uchino's results have shown that because of interposed inhibitory neurons, their simultaneous stimulation acts to enhance the response to the GIF in a manner analogous to the enhancement to angular acceleration by bilateral inhibitory interaction in processing of semicircular canal neural information (18–20). In the semicircular canal system this is called mutual commissural inhibitory interaction and it has been shown to enhance the neural response of single VN neurons to angular acceleration (19) (see Figure 3). In the following I show how Uchino's results apply in the otolithic system, but some general features of vestibular afferents and physiological conventions in this area need to be clarified.

### Sustained and Transient Otolithic Systems

As well as providing information about the direction of the GIF, the afferents from each otolithic macula provide information about different temporal aspects of the stimulus. As a simplification these different neural channels are characterized as the sustained and the transient systems (14, 21, 22). They are most likely extremes of a continuum.

Afferents arising from the striola which have irregular resting activity and constitute the origin of the transient system—they prefer high frequency GIF stimuli and are activated by sound or vibration. In the otolithic maculae the striola is a comparatively thin band with a small number of receptors and afferents, and it is afferents from this band which respond to vibration [see (23) for a review]. The thin band can be seen by inspection of whole mount preparations of the maculae (14, 24). There are many more receptors and afferents in the extra-striolar area, and afferents from the extra-striolar area have regular resting discharge and constitute the sustained system. The sustained afferents prefer maintained or low frequency GIFs stimuli and do not respond to sound or vibration at physiological levels (22, 25–27). Afferents in the two systems have different responses to stimulus onset, different thresholds for activation by electrical stimulation and different adaptation rates to maintained stimulation (6). This differential receptor and afferent organization of the otoliths is analogous to the organization of the retina with 1,000,000 cones concentrated at the fovea specialized to detect fine detail but 125,000,000 receptors in the rest of the retina (28).

In the present paper the focus is on the sustained system since diagrams in Uchino's papers show that his results probably originated mainly from isolated electrical stimulation of the extra-striolar macula areas (29, 30) from where the sustained afferents mainly originate. The physiology of the transient system and the clinical testing of it have been extensively reviewed recently (1, 21, 23, 31, 32) and so they will be covered only briefly in this paper.

### Otolith Physiology—General

The opposite polarization of receptors across each macula is puzzling since a GIF directed across the utricular macula will excite receptors on one side of the LPR and simultaneously act to disfacilitate (silence) receptors on the opposite side of the LPR (Figure 2B). It would seem the two neural signals from the one utricular macula should cancel. In fact, detailed physiology in the VN show exactly the opposite—that instead of canceling, the simultaneous stimulation of the oppositely polarized hair cells, both in the saccular macula and the utricular macula, enhances the otolithic response to the GIF similar to the enhancement shown above for the semicircular canals. Uchino called this phenomenon cross-striolar inhibitory interaction. In the case of the utricular macula this enhanced response is further complemented by inhibitory interaction between the two labyrinths which Uchino called commissural inhibitory interaction (29). Below I discuss how cross-striolar inhibition works and then I address commissural inhibitory interaction. The following shows how Uchino's results operate in the VN, using schematic figures derived from Uchino's representations. These patterns of response organization were shown by intracellular recording of single neurons in the VN and measuring their response to isolated electrical stimulation of distinct locations on each otolithic macula and measuring the excitatory or inhibitory responses in VN neurons to such stimulation [summarized in (29, 33)].

The naming convention used to describe the response of central semicircular canal neurons is used here to describe otolithic neurons. Specifically type I neurons are excitatory neurons in the VN receiving monosynaptic afferent projections from primary otolithic afferent neurons and having multiple central projections. Type II neurons are inhibitory neurons in the VN which are activated by an axon branch from a type I neuron projecting to, and so inhibiting, other type I neurons. The schematic figures [redrawn from the schematic figures used by Uchino (29, 33, 34)], depict exemplars of these neural types and their established connections to show how these neural types are activated and interact. These principles operate for a limited number of otolithic neurons—many otolithic neurons are outside the interactive “loops” described below.

### Cross-Striolar Inhibition in the Saccular System

**Figure 4:**
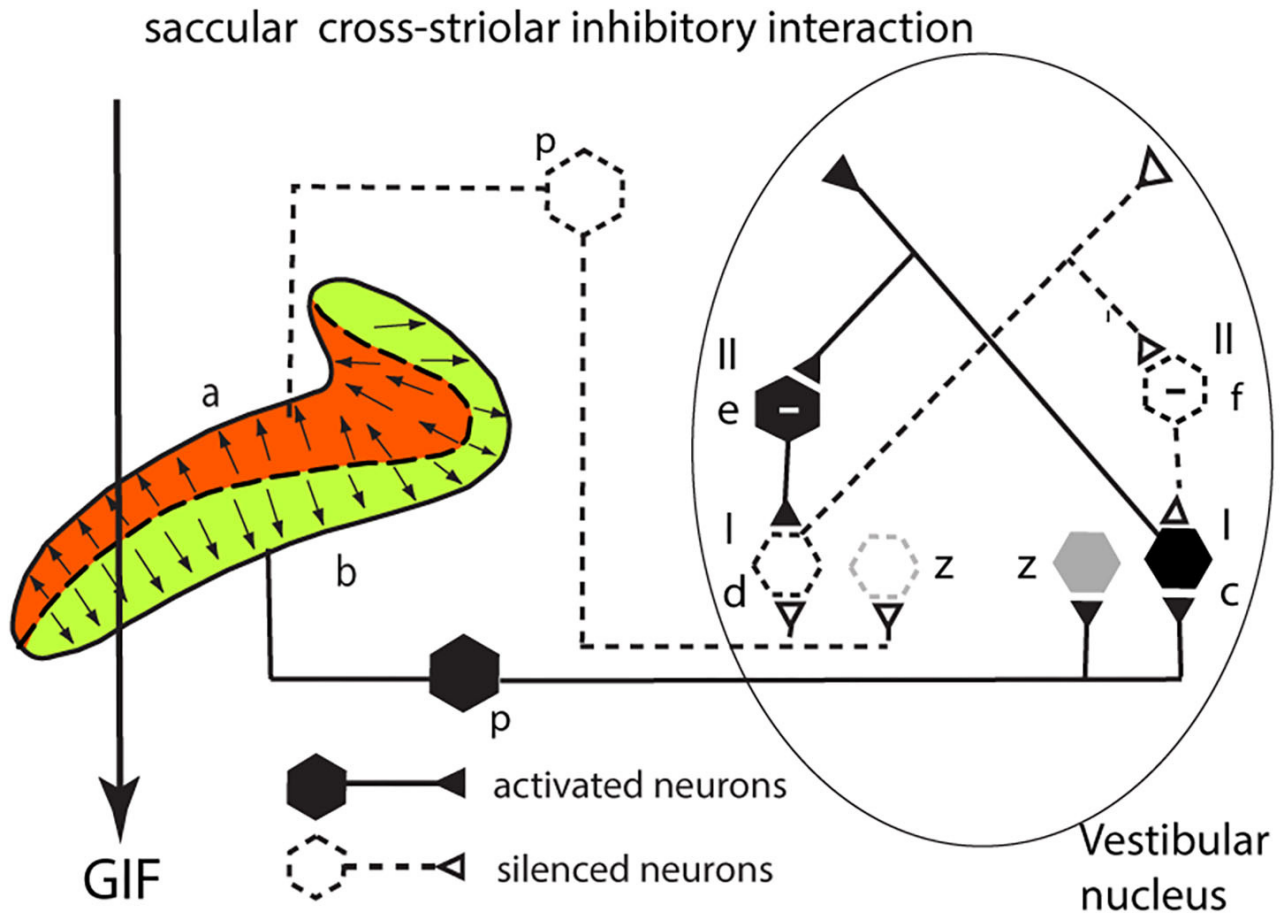
Cross-striolar inhibition in the saccular macula—see text for full explanation.

This section explains how cross-striolar inhibitory interaction works within each saccular macula. Figure 4 shows a schematic representation of the saccular macula with receptors projecting to the VN. Receptors in the ventral sector (b) are activated by the GIF—the force of gravity (thick arrow)—and facilitate the primary afferent neurons (p) which in turn project to and activate the neuron in the VN labeled type I (c) whose firing rate accordingly increases. Simultaneously receptors in the dorsal sector of the saccular macula (a) are deflected away from the kinocilium, so they disfacilitate their primary afferent neurons (dashed lines) and so disfacilitate the type I neurons in the VN labeled d. An axon branch from the facilitated type I neuron (c) projects to an inhibitory VN neuron [type II (e) shown with a—sign] which inhibits the VN neuron receiving input from the dorsal sector (d)—further silencing this disfacilitated neuron. In turn this disfacilitated VN type I neuron exerts less drive to the inhibitory type II neuron (f) which exerts less inhibition on the activated type I neurons. Less inhibition from (f) is equivalent to activation of (c)—this release from inhibition is called disinhibition. So, the type I neuron (c) receives both direct excitation from the ventral sector of the saccular macula and also additional excitatory drive by disinhibition from receptors in the dorsal sector. The outcome of the cross-striolar inhibitory interaction is an enhanced neural signal with the difference in firing between the two opposing sectors being greater than would be the case without the inhibitory interaction. So instead of canceling, the effect of the cross-striolar inhibitory interaction between the oppositely polarized sectors is to enhance the neural response to the stimulus. Once again it should be noted that this “closed loop” is only part of the story—the afferents from each sector project to other VN neurons outside this loop (z) which are grayed out in this and the following figures.

Uchino referred to this whole process as cross-striolar inhibition, and it applies in both saccular and utricular maculae. He reported that more than 61% of saccular neurons tested in the VN showed cross-striolar inhibitory interaction, but cross-striolar inhibitory interaction is not as widespread in the utricular macula (it was only seen in 30% of utricular neurons tested) (29, 30). The two saccular maculae function largely independently since there is virtually no bilateral interaction between the two saccular maculae in each labyrinth—no commissural inhibition (34) —whereas utricular neurons receive commissural inhibition (as shown below) as well as this cross-striolar inhibition (30).

### Cross-Striolar Inhibition in the Utricular System

**Figure 5:**
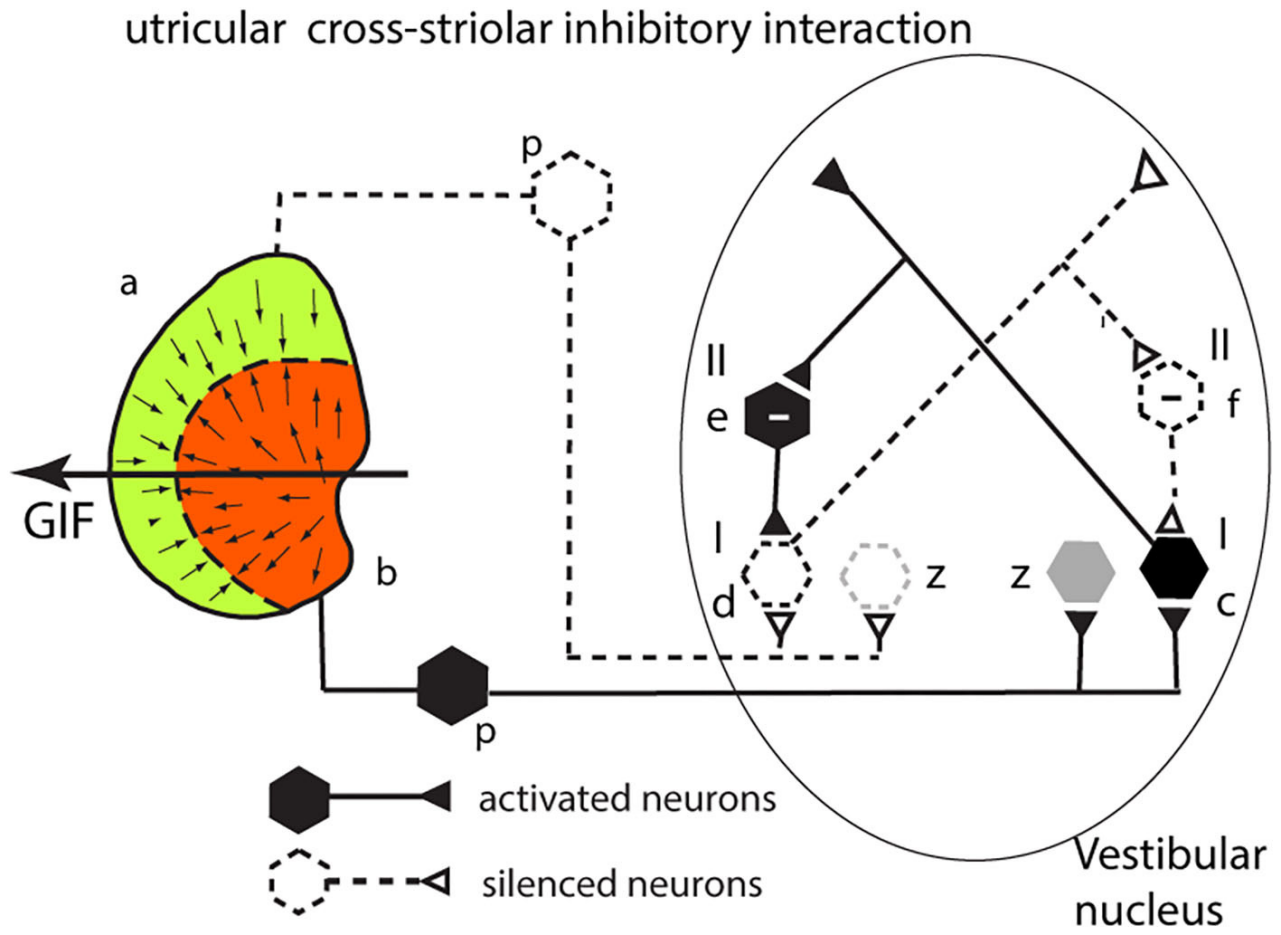
Cross-striolar inhibition in the utricular macula—see text for full explanation.

The analysis for the utricular macula is identical to that given above for the saccular system. Consider a GIF stimulus directed across the left utricular macula from right to left (Figure 5). It will activate receptors in the left medial sector which project to and activate a type I neuron in the VN (c). Simultaneously receptors in the left lateral sector (a) will be disfacilitated. These project to a type I in the VN (d), and so its activity will be reduced. An axon branch from the facilitated type I (c) projects to an inhibitory (type II) VN neuron (e shown with a—sign) which inhibits the VN type I neuron (d) receiving input from the lateral sector—further silencing the disfacilitated neuron. In turn this disfacilitated VN type I neuron (d) exerts less drive and so less inhibition via the type II neuron (e) on the activated type I neuron (c). So, the activated type I neuron (c) receives both direct excitation from the medial sector of the utricular macula and additional excitatory drive by disinhibition from receptors in the lateral sector. The outcome of the cross-striolar inhibitory interaction is an enhanced neural signal with the difference in firing between the two sectors being greater than would be the case without the inhibitory interaction. In summary: in both the utricular and saccular maculae, cross-striolar inhibitory interaction serves to enhance the response to the GIF stimulus.

### Commissural Inhibition in the Utricular System

#### Medial Sectors

**Figure 6:**
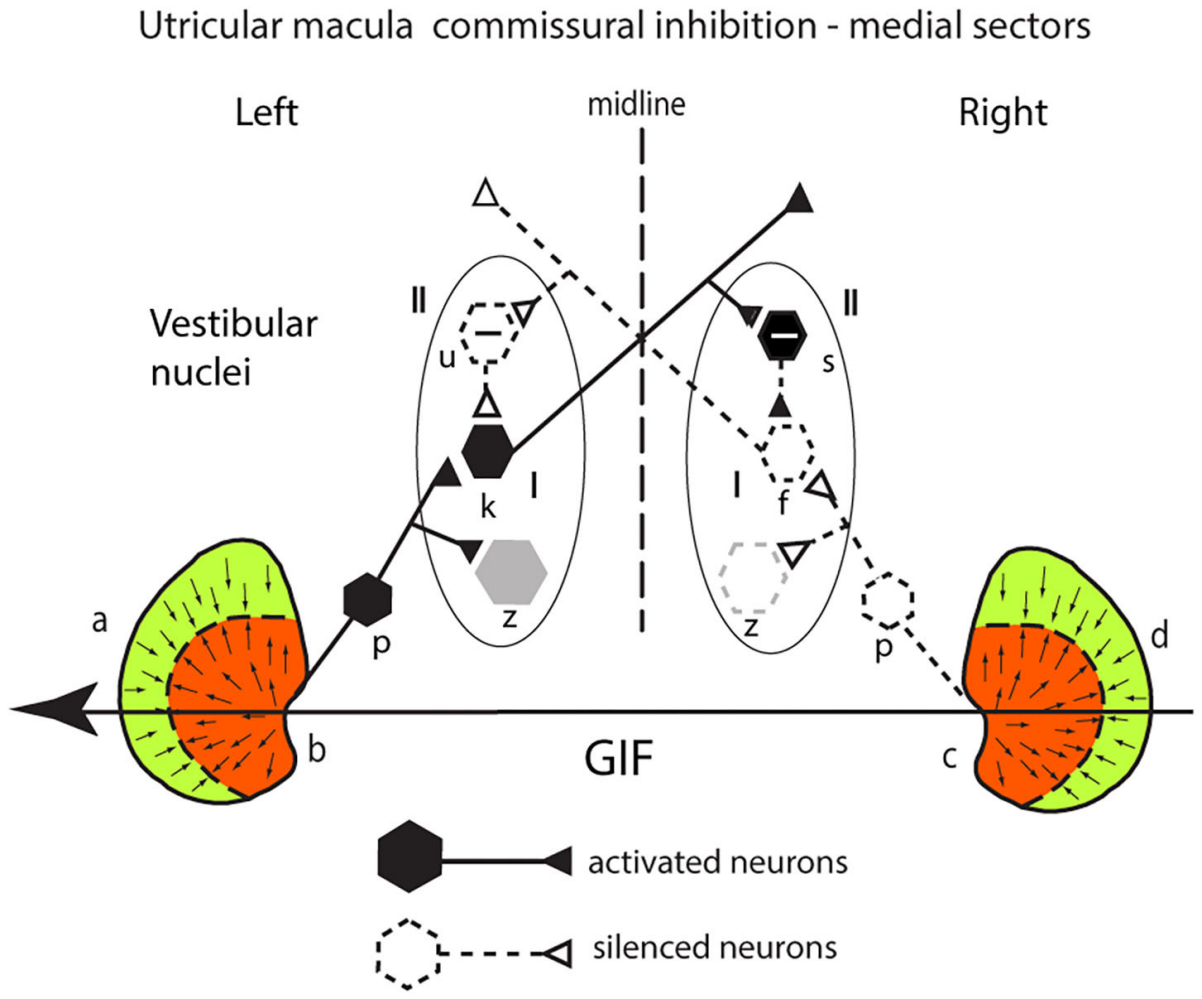
Commissural inhibition in the utricular macula—see text for full explanation.

The utricular system also receives an additional enhancement due to inhibitory interaction between the two labyrinths which Uchino called commissural inhibitory interaction (30). Consider a GIF directed from right to left across a subject's head, during a roll head tilt to the left [see the schematic representation of both utricular maculae (Figure 6)]. This stimulus will activate (facilitate) receptors in the medial sector of the left utricular macula (labeled b) because the direction of the stimulus is aligned with the preferred directions of medial sector utricular receptors on the left. It will simultaneously act to disfacilitate receptors in the medial sector of the contralateral right utricular macula (labeled c) because the direction of the stimulus is opposite to the preferred direction of receptors in the right medial sector. The afferents from these excited left medial sector receptors project to and activate neurons in the ipsilateral (left) VN (type I neurons) (labeled k in Figure 6). The axon of that neuron projects to a contralateral inhibitory type II neuron (s) on the right side, increasing its firing and so increasing the inhibition exerted by s onto the type I neuron (f) in the right VN which is receiving disfacilitated afferent input from the medial sector of the right utricular macula. These afferents and type I neurons on the right are already firing at a reduced firing rate (shown as dashed lines) since the stimulus direction is opposite to their preferred direction, and so the stimulus itself is acting to disfacilitate the receptors and afferents. In turn the reduced firing of the right sided type I (f) will reduce the inhibition from the left side via the inhibitory neuron type II (u) acting on the left type I (k) allowing it to fire at an even higher rate (disinhibition).

The outcome is that the stimulus—a roll head tilt left ear down—will cause an imbalance in neural activity between the two VN—a high firing rate for type I neurons on the left and a low firing rate for type I neurons on the right. In this way, commissural inhibitory interaction between the corresponding medial sectors on each side acts to enhance the imbalance in the neural activity in response to the stimulus. Once again, the otolithic neural response depends on two sources of facilitation—direct facilitation from the ipsilateral activated otolithic receptors and reduced inhibition (disinhibition) originating from the contralateral disfacilitated receptors. Again, other VN neurons (gray) are outside this loop.

### Commissural Inhibition in the Utricular System

#### Lateral Sectors

**Figure 7:**
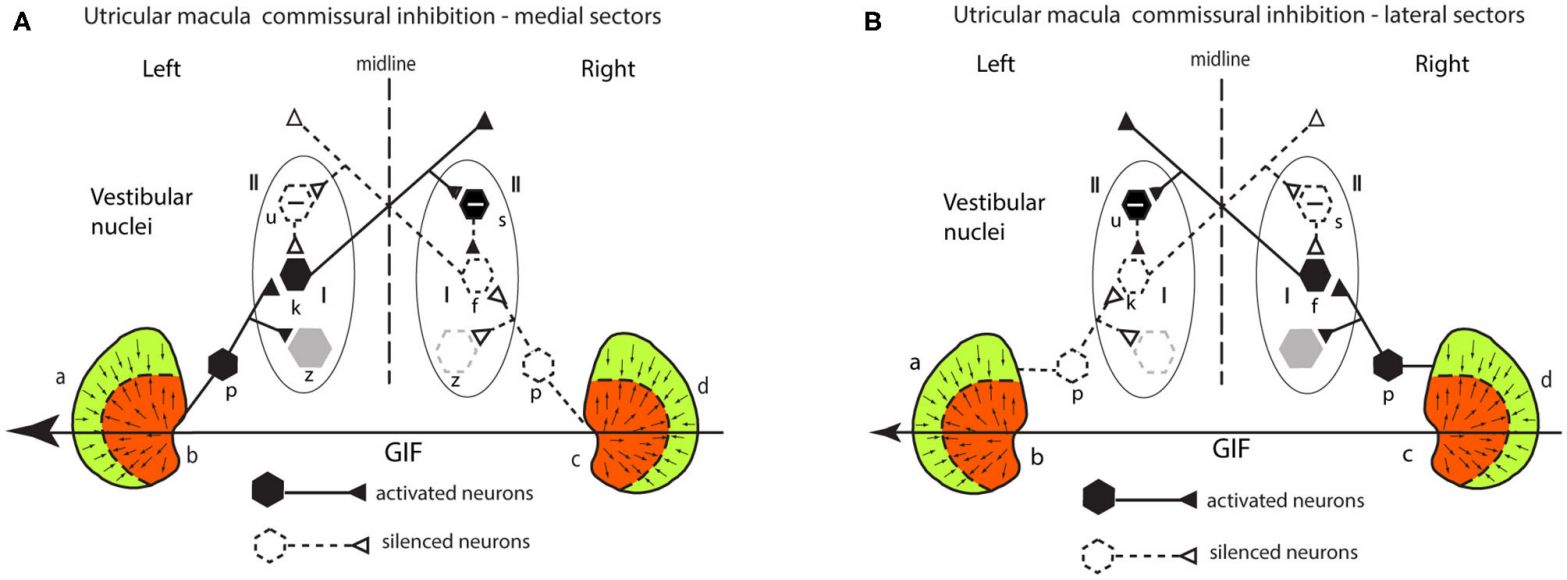
**(A,B)** Schematic representation of the two utricular maculae and examples of afferent neurons projecting from the medial sector of each macula **(A)** and lateral sectors **(B)** of each utricular macula to synapse on central excitatory otolithic neurons (hexagons) in the VN. The figure shows the commissural interaction in the medial **(A)** and lateral **(B)** sectors.

There is comparable mutual inhibitory interaction in the VN between afferents from the two lateral sectors of the utricular macula (a and d) (Figure 7), but now for the same GIF directed from right to left it is the right lateral sector which is activated and the left lateral sector which is disfacilitated. Inspection of response of the medial and the lateral circuits in Figure 7 raises the question—why doesn't the right lateral sector activation simply cancel out the left medial sector activation so there is no imbalance in activity between type I neurons in the two vestibular nuclei? Don't they just cancel centrally? The following are three reasons that the response from the left medial sector predominates:

1. the area of the medial sector (and so the number of afferents) is larger than the area of the lateral sector (35).
2. cross-striolar inhibition favors the medial sector (60% of neurons tested) vs. lateral sector (30% of neurons tested) (30).
3. commissural inhibition is more frequent for medial sector afferents (56%) than for lateral sector afferents (44%).

So, taking all of this together, the left medial response is larger because both cross-striolar and commissural inhibition favor the medial sector. Confirmation of the medial sector predominance comes from recordings of VN neurons to roll-tilt, which shows more VN neurons activated by medial sector stimulation (48% by ipsilateral ear down tilt) than are activated by lateral sector stimulation (26% contralateral ear down tilt) (36).

A final consideration is that anatomical evidence indicates that the medial and lateral sectors of the utricular macula have different projections. Both project to the brainstem and cerebellum (37) but the lateral sector projection to the cerebellum is greater.

## Otolith Stimulation and Responses

This section relates the basic neurophysiology of the peripheral otolith system to potential clinical tests of otolithic function by gravity or low frequency linear acceleration and so testing predominantly the sustained system. The effect on otolith function by such procedures as unilateral vestibular loss, selective otolith ablation, and galvanic stimulation on responses in sensory, oculomotor and postural control systems are discussed. There are extensive literatures about the response of each of these systems to otolithic stimulation or manipulation, and here I note general principles which are of interest for the development of clinical otolithic tests, rather than presenting an exhaustive review. Most clinical tests of otolith function have focused on measuring eye movements to otolithic stimulation, in particular to stimuli such as roll-tilt. Whilst ocular torsion has usually been measured it is important to note that each quadrant of the utricular macula projects to different eye muscles (38).

### Tests of the Sustained System of Otolith Function

#### Responses to Roll Head Tilt in Healthy Subjects and After Unilateral Vestibular Loss

At rest the central otolithic neuronal signals in the VN from the bilateral medial utricular maculae are presumed to be in equilibrium. However, stimulation or unilateral loss will upset that balance and generate responses. The oculomotor response to roll-tilt consists of mainly ocular torsion (also called counterrolling—OCR). In response to the increasing lateral GIF across the utricular macula as the head rolls, the eyes roll with the upper poles of both eyes being displaced in the orbit in a direction opposite to the GIF (39–44). The OCR is usually a very small fraction of the roll-tilt angle (about 8–10 deg maximum in healthy people). Increasing the roll-tilt stimulus systematically increases the magnitude of the GIF vector across the medio-lateral sector of the maculae and so progressively increases the neural imbalance between utricular neurons in the two nuclei. As roll-tilt increases there is a non-monotonic increase in OCR which is likely mainly due to utricular stimulation, although there is evidence for a small contribution to OCR from the saccular macula at large roll-tilt angles (45, 46). Direct electrical stimulation of the utricular nerve in cats (47) caused torsion of both eyes with the upper poles of the eyes rolled away from the side being electrically stimulated. This torsion occurred primarily because of utricular activation of the contralateral inferior oblique and ipsilateral superior oblique muscles (48). Additionally, there were small horizontal and vertical components.

**Figure 8:**
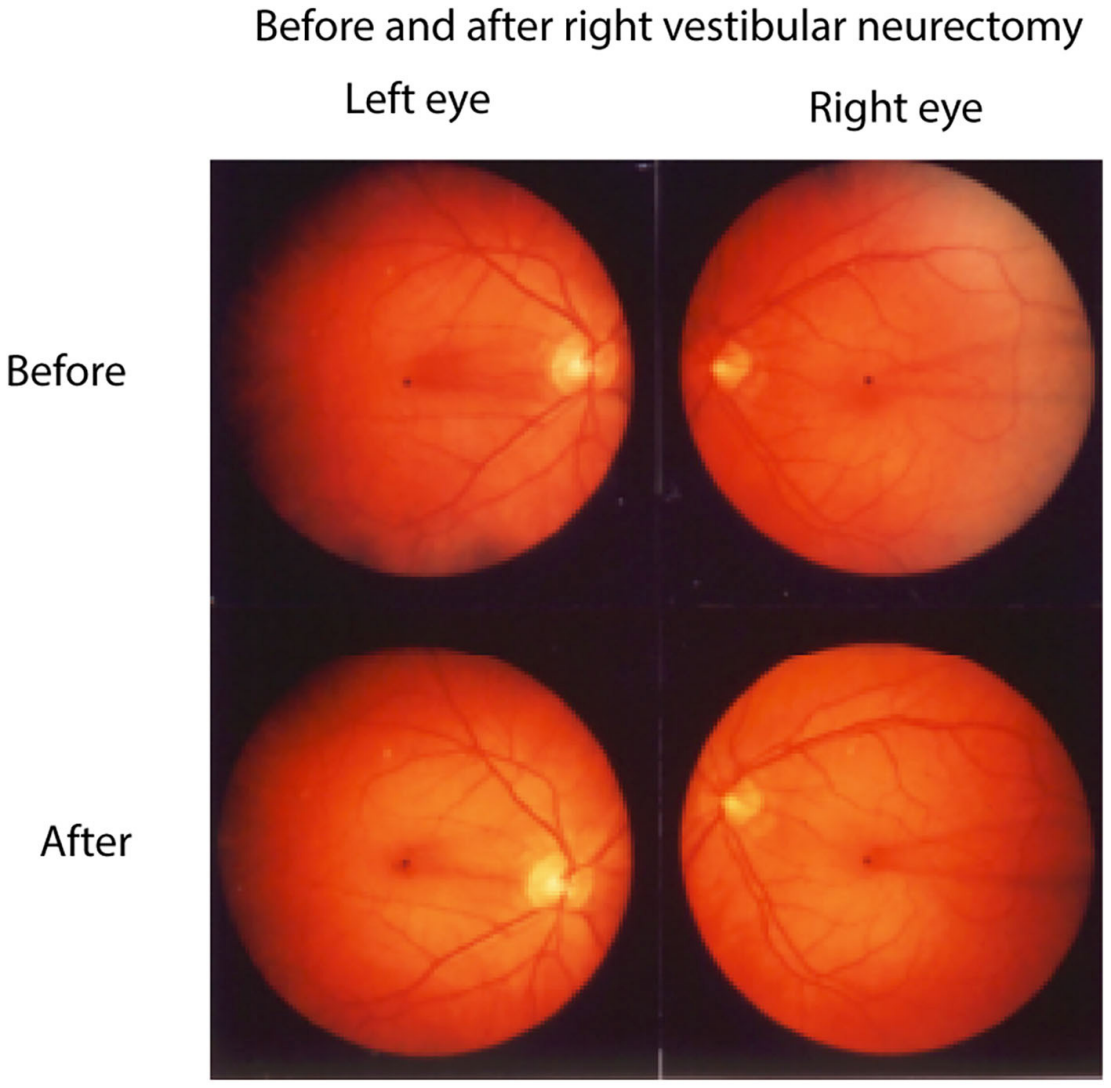
Fundus photographs of the left and right eyes of a patient before and 1 week after a neurectomy of the right vestibular nerve showing the unique pattern of retinal blood vesels in each eye. The blood vessel patterns show very clearly that after unilateral vestibular neurectomy both eyes adopt a maintained roll eye position, rolled toward the patient's lesioned (right) side. Reprinted by permission from Springer Nature, Curthoys et al. (49) © 2020.

Complementing the results of increasing roll-tilt stimulation is the evidence that unilateral section of the vestibular nerve causes both eyes of human patients to adopt a maintained rolled eye position (49–51), rolled toward the operated side (Figure 8). This result follows from the physiological analysis above. In a healthy individual if one labyrinth is suddenly silenced, for example by surgical removal or severe neuritis then the equilibrium between the two VN is lost with otolithic type I neurons in the VN on the lesioned side being silenced and otolithic type I neurons on the intact side presumably having normal resting activity. Such an imbalance corresponds to the utricular neural response to roll head tilt to the healthy side which causes a small OCR toward the opposite (lesioned) ear. Such an imbalance of utricular otolithic neural activity corresponds to the imbalance of semicircular canal neural activity after unilateral loss in the semicircular canal system (52–54). Acutely in the case of the semicircular canals the imbalance results in nystagmus and vertigo. Acutely, in the case of the utricular maculae the imbalance is equivalent to a large roll-tilt and drives the head and eyes to roll toward the lesioned side and to maintain this rolled position (49, 55). There are simultaneous postural changes—head tilt to the affected side, falling to the affected side. This loss-induced torsion and postural change reduce over time in the process called vestibular compensation (20). It is argued that this maintained ocular torsion position is probably an otolithic response rather than a canal response because canal loss induces a change in eye velocity (nystagmus) rather than a maintained eye position and because isolated loss of the utricular macula with canals intact in guinea pigs caused similar responses (56).

**Figure 9:**
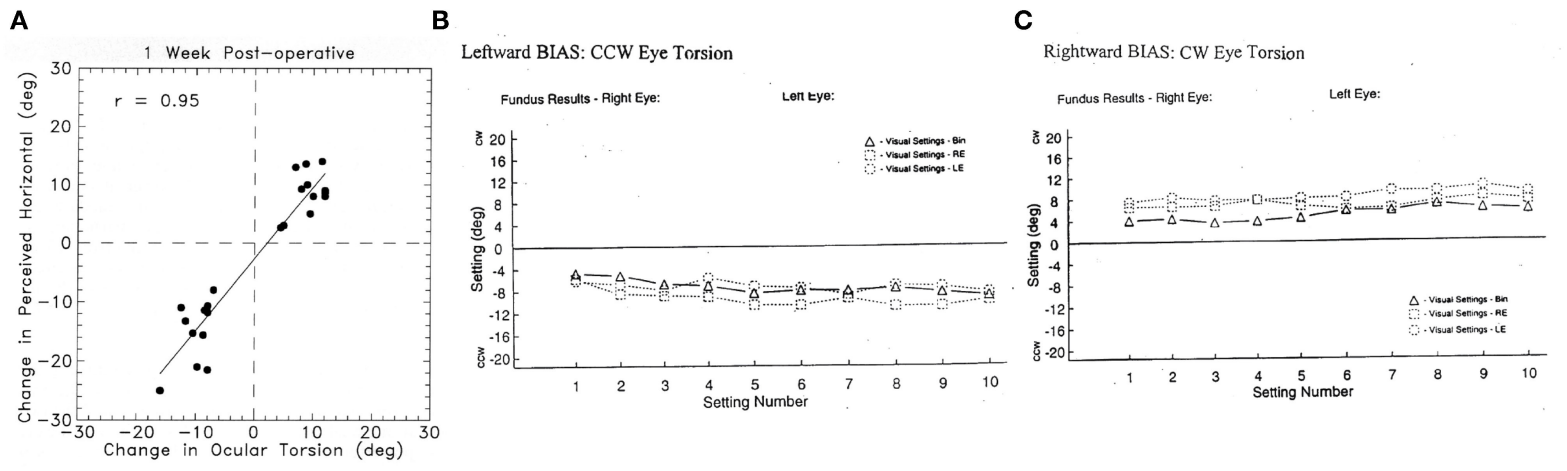
**(A)** To show the very close relationship between the change in ocular torsional position after unilateral vestibular neurectomy and the corresponding change in the setting of a visual line to the subjective visual horizontal (49) in patients after unilateral vestibular loss. This setting is called the visual bias test. This close correspondence justifies the use of the perceptual result to indicate the torted position of the eye. Reprinted by permission from Springer Nature, Curthoys et al. (49) © 2020. **(B)** Results of the visual bias clinical test of otolith function in a patient with a left side loss. The patient made 30 successive settings to where they judged the gravitational horizontal to be: 10 settings with binocular viewing (BIN), 10 with left eye only (LE), and 10 with right eye only (RE). CCW, counterclockwise from the patient's point of view (i.e., the line is set so the left side is below the true gravitational horizontal); CW, clockwise from the patient's point of view. The results show a very consistent shift of the perceived horizontal so after left unilateral loss, the visual bar is set left side down from the patient's point of view, which corresponds to the rolled horizontal meridian of the eye, as shown in Figure 8. **(C)** Similar results from a patient with a right-side vestibular loss which causes the visual line to be set so that the right side of the line is set down, corresponding to the rolled horizontal meridian of the eye. Most subjects and patients can perform this task with very small variability as shown here (Settings consistently >2° are outside the normal range).

In patients weeks or months after unilateral vestibular loss there remains a small ocular torsion which is usually only a few degrees, but it causes a small systematic bias of the perceived orientation of a horizontal (or vertical) visible line in an otherwise darkened room so that the line no longer appears to be horizontal (or vertical) (20, 49, 57) (Figure 9). This perceptual error occurs because the ocular torsional position is rolled by a few degrees toward the affected ear and the orientation of the retina is a major determinant of visually perceived vertical or horizontal in an otherwise darkened room (43). This small perceptual error is called the visual bias (49, 58, 59). It occurs with horizontal lines [subjective visual horizontal (SVH)] or vertical lines [subjective visual vertical (SVV)]. Although they are not large, the ocular torsion position angle and the visual bias appear to be an almost permanent legacy of probable otolithic origin after unilateral vestibular loss (49, 60). Over time this ocular torsion and the visual bias decrease but never completely vanish. The visual bias is a simple useful clinical indicator of asymmetric sustained otolithic function (49, 61, 62) (Figure 9). Why does the maintained roll of the eye persist? It appears that the reduced afferent input from the affected utricular macula results in permanently slightly reduced neural input projecting to the ipsilateral superior oblique and contralateral inferior oblique eye muscles so that the eye adopts a rolled eye position—rolled toward the affected ear. It should be noted that maintained ocular torsion is not necessarily a specific indicator of peripheral otolithic loss—it can occur with central lesions along the pathway from the otolithic receptors to the eye muscles (63). Ocular pathology can also cause changes in SVV.

One other otolith oculomotor response after unilateral peripheral vestibular loss is skew deviation which refers to vertical misalignment of the two eyes after unilateral vestibular loss with the ipsilesional eye being lower in the orbit than the contralesional eye. This is usually a very small effect which can be identified clinically by alternately covering each eye and identifying if a vertical refixation is needed. However, lesions of central vestibular pathways also generate skew (63).

Acutely after unilateral vestibular loss the OCR response to maintained roll-tilt stimulation shows a temporary reduction in OCR for roll-tilts to the affected ear (64, 65), but testing OCR to roll-tilt in chronic patients weeks after unilateral loss shows there is no asymmetry of OCR—it fails to identify which side is affected (7, 40, 66, 67). The empirical result is that measuring OCR to left and right roll-tilt does not reliably indicate the affected side after unilateral loss in chronic patients, whereas the visual bias shows the affected side in acute and chronic patients. It appears that vestibular compensation is acting to nullify the initial asymmetrical OCR response.

#### Unilateral Centrifugation

Another way of generating sustained GIF stimulus depends on the fact that the two otoliths are around 3.6 cm from the midline of the head (68, 69). As a result, a constant velocity rotation of a patient on a rotating chair with the center of the head positioned exactly over the axis of rotation induces a GIF across each utricular macula. This is called unilateral centrifugation (70, 71). At high rotational velocities (e.g., 300 deg/s) this GIF achieves a reasonable magnitude but in healthy people being directed outward, it is opposite in each labyrinth, so the effects of the opposite GIFs cancel, and no systematic torsion occurs (71). However, if the subject's head is displaced 3.6 cm laterally so that one labyrinth is exactly over the axis of rotation, the GIF during high velocity rotation [300 deg/s at 7 cm generates about 0.2*g* laterally (70)] and so stimulates the utricular macula in the “off-axis” ear causing torsion and perceptual responses, so the utricular function of that ear can be measured (70). This unilateral centrifugation test shows unilateral loss both acutely and in chronic patients (70–72). The rotational velocities required are very high (around 300 deg/s and so potentially dangerous), the resulting torsion is small (just a few degrees), variable between patients (71) and difficult to measure so this method has not proved to be a widely used practical clinical test of unilateral otolith function.

#### Oculomotor Response to Linear Translation

**Figure 10:**
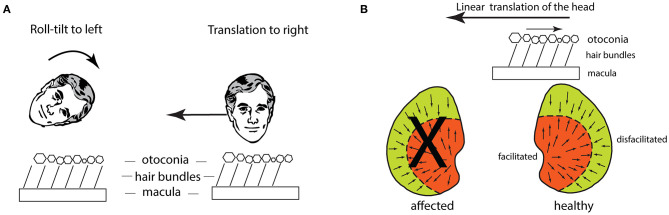
**(A)** Schematic figures to show how the hair bundles of utricular receptors are deflected identically for a roll-tilt left ear down and a horizontal head translation to the person's right. **(B)** The effect of lateral linear acceleration toward the affected ear in a patient with unilateral vestibular loss (the X shows the affected utricular macula). The linear acceleration directed toward the affected ear causes the otoconia to remain in place because of inertia and so the hair bundles of all the utricular receptors are deflected opposite to the linear acceleration. This direction of hair bundle deflection is an excitatory stimulus for receptors in the medial sector of the utricular macula, and an inhibitory stimulus for receptors in the lateral sector. The medial excitation would be expected to generate a compensatory horizontal eye velocity response, however in human patients there is instead a (temporary) reduced horizontal eye velocity response for linear accelerations directed to the affected ear.

A lateral translation of the head causes deflection of the utricular receptor hair bundles because of inertia. The otoconia, attached to the upper surface of the otoconial membrane, tend to stay in place and so drag the hair bundles of utricular receptors opposite to the lateral translation (Figure 10A). In this way lateral translation should cause OCR opposite to the translation direction, and that result has been reported in humans (73) and chinchillas (74). There were also small horizontal eye movements—lateral translation to the left causes both eyes to move horizontally to the right (75, 76), depending on many factors such as fixation direction and distance (77). This compensatory horizontal eye movement response is due in part to utricular afferents which project to ipsilateral abducens nucleus (33, 78, 79), but other cerebellar pathways probably contribute (77).

This horizontal eye movement to lateral linear acceleration (80) has been used to try to identify the unilateral utricular loss, by analogy with the success of the horizontal eye movement to angular acceleration identifying the side of unilateral semicircular canal loss (81). A horizontal angular acceleration toward the affected ear results in a reduced horizontal compensatory eye velocity response and so permanently identifies the affected semicircular canal (82). That result occurs essentially because of the uniform receptor organization on the crista in each horizontal canal, and the projections from the canals to the contralateral abducens nuclei (20). Corresponding to that canal result is the evidence that acutely after unilateral loss linear head translations toward the affected ear cause reduced compensatory horizontal eye movements (80). But in contrast to the permanent reduced response for angular acceleration stimulation of the affected canal, the reduced horizontal eye velocity response for ipsilesional lateral accelerations is very short-lived and testing 6 weeks after loss, shows there is no detectable reduction in the horizontal eye velocity to lateral translation toward the affected ear (80). Two other factors should be noted: the whole body lateral linear accelerations were very small stimuli—about 0.24 *g* (80), with very long rise time (so jerk was small), and had a very long latency around 35–45 ms (80) compared to a latency of about 7 ms for the horizontal eye velocity response to semicircular canal stimulation (82, 83).

Whilst the reduced horizontal eye velocity for linear accelerations toward the affected ear seems consistent with the reduced horizontal eye velocity response for angular acceleration of the canal after unilateral loss—both giving reduced horizontal eye movement responses for stimuli directed to the affected side—the result for linear accelerations after unilateral loss in fact is not readily explicable by the receptor organization of the utricular maculae and their neural projections! This conundrum is shown in Figure 10B—the linear acceleration of the head toward the affected ear causes the otoconia to remain in place because of inertia, so the hair bundles of the receptors on the remaining utricular macula are deflected opposite to the direction of the linear translation. As shown in Figure 10B, because of their respective polarizations, that means that receptors in the medial sector of the healthy macula are excited, while those in the lateral sector are inhibited. However, excitation of the medial sector receptors should cause an increased oculomotor response (as it does for the torsional response to lateral roll-tilt of the head) but the empirical result for horizontal eye velocity to lateral linear accelerations in patients is exactly the opposite—the linear acceleration toward the affected ear causes a reduced compensatory horizontal eye movement response (80). To accommodate this puzzling result, Lempert suggested that it must be receptors in the lateral sector of the utricular maculae which generate compensatory horizontal eye velocity responses to lateral linear accelerations (80). In the case of unilateral loss, the receptors in the lateral sector would be inhibited by the lateral linear acceleration stimulus to the affected ear and so their inhibition would cause a reduced horizontal eye movement response, as is observed. Further compounding the puzzle is the fact that the projections from the utricular macula to abducens are from utricular macula to ipsilateral abducens nucleus (78, 79) so utricular activation would generate an ipsilateral eye rotation for ipsilateral utricular stimulation (23, 84), although the precise origin of these projections from the utricular macula (medial or lateral sectors) to the ipsi lateral abducens is unknown. We can best summarize the story of lateral linear accelerations by noting the logical and empirical problems which the results of linear acceleration on horizontal eye movements after unilateral loss have shown (77, 85). This puzzling result has not been explained. The long latency for the horizontal component to lateral linear accelerations suggests the involvement of indirect pathways, such as via the cerebellum. Indeed, Maklad et al. have shown that afferents from the two sectors of the utricular macula have different projection patterns—in the mouse afferents from the medial sector project mainly to the brainstem and afferents from the lateral sector project mainly to the cerebellum (37).

In summary clinical tests based on asymmetry of ocular responses to roll-tilt or to lateral translation do not provide a reliable indicator of the side of unilateral otolithic loss in long term patients, so using these tests, the clinician cannot reliably determine whether the left or right utricular macula has been compromised. The visual bias test and unilateral centrifugation are indicators of sustained otolith function which do show the affected side in chronic patients.

#### Tests of the Transient System of Otolith Function

Returning to the peripheral otolithic sense organs—each otolithic macula contains a band, a stripe, around the LPR and the band is called the striola. In this band the receptors and afferents are structurally and functionally specialized. The hair bundles are shorter and stiffer than those in the extrastriola area and they are only tenuously attached to the otolithic membrane [see (9, 14, 23)]. Recordings from primary afferents originating from this region with irregular resting activity show they are activated and are even phase locked up to high frequencies of sound and vibration (e.g., 500 Hz and up to 3,000 Hz) (14, 22, 26, 86). That result means that each otolithic sense organ has two modes of responding—for low frequency GIFs and low frequency vibration the otolithic macula responds as an accelerometer, but for high frequency stimuli it responds as a seismometer (23, 83, 85, 86). How can that dual mode of responding occur? At low frequencies the otoconia and hair bundles move relative to the receptor cell body, but at high frequencies the receptor cell body moves relative to the otoconia and hair bundles. In both cases the hair bundles are deflected relative to the receptor cell body, so the receptor is activated, but the dynamics of that deflection are completely different (87).

**Figure 11:**
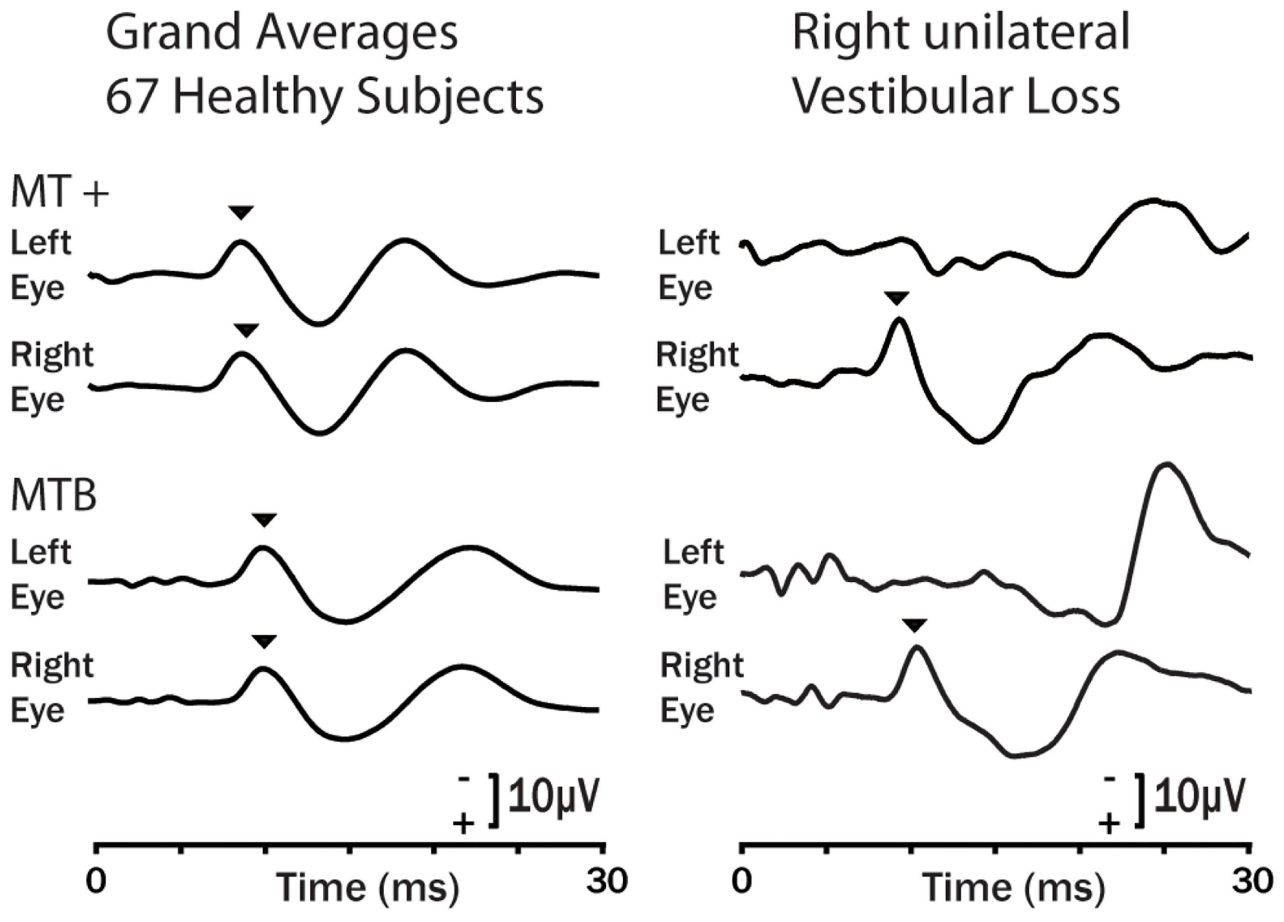
Examples of the oVEMP response of healthy subjects (grand means left column) and a typical patient with right side unilateral vestibular loss in response to bone conducted vibration stimulation (92) on the forehead at Fz using the 4810 Mini-Shaker driven by a condensation click (MT +) and 6 ms tone bursts at 500 Hz (MTB). Bone conducted vibration delivered at Fz stimulates both ears about equally and results in small symmetrical oVEMP n10s (arrow heads) beneath both eyes (93). The response is similar for a single tap (MT +) or a brief tone burst (MTB). For the patient the asymmetrical response is clear: the n10 component of the oVEMP (arrowhead) is absent under the eye ***contralateral*** to the patient's affected ear because the oVEMP is a crossed response (90). Copyright © 2008, Karger Publishers, Basel, Switzerland.

The response of the otolithic receptors to sound and vibration seems at odds with what is regarded as the usual response of otoliths to gravity and low frequency linear acceleration. Evolution provides an insight. Fish do not have cochleas but have otolithic maculae. Fish primary otolithic neurons are activated by to vibration and show precise phase locking up to high frequencies (88) and show directional tuning (89). It appears that these features of otolithic processing have been transferred to mammals. This high frequency mode is particularly important since the myogenic responses triggered by the high frequency activation of these receptors at the striola do show unilateral otolithic loss in both acute and chronic patients (90) which is in sharp contrast to the failure of the low frequency otolithic stimuli (testing mainly the sustained system) to detect the affected side as we have shown. The two main measures of the transient system are short latency vestibular evoked myogenic potentials (VEMPs)—the ocular VEMPs from beneath the eyes recording primarily utricular functional status and the cervical VEMPs recording primarily the saccular functional status (2, 21, 87, 91) (Figure 11). The evidence for the ability of VEMPs to detect unilateral loss was shown first by Colebatch and Halmagyi for cVEMPs (94) and Iwasaki et al. for oVEMPs (90), and both results have been confirmed in many studies since, so that these tests are now standard clinical tests of unilateral otolith function as is covered in the recent reviews devoted to VEMPs noted above.

The sustained and transient modes of otolithic operation have an interesting consequence—that there could be a dissociation between the results of the low frequency and high frequency tests. That has been confirmed in healthy subjects by Zalewski et al. (95) who found no correlation between ocular torsion (bias) (low frequency) and oVEMPs (high frequency), indicating the two types of tests are probing different functions. In the case of patients, complete unilateral loss of otolith function abolishes both high and low frequency responses, but in other patients, one or the other of the high frequency or low frequency response modes could be affected whilst leaving the other mode intact. Cherchi has reported exactly this dissociation between tests of sustained and transient utricular function after vestibular neuritis (96). It will likely also occur in patients after treatment with the ototoxic antibiotic gentamicin which preferentially attacks the type I receptors at the striola (97, 98) and so would degrade the transient system but leave the sustained system functioning. It would be expected that some such patients would have reduced or absent oVEMPs (transient function) but preserved ocular counterrolling to roll-tilt stimulation (sustained function).

In summary, Short latency myogenic responses to sound or vibration stimulation of the otoliths do show clinically important clear permanent response asymmetries after unilateral vestibular loss, due to loss of the transient system originating from receptors at the striola of the utricular and saccular macula.

#### Effect of Isolated Otolithic Macula Loss

In animal studies it has been possible to carry out selective lesions restricted to the utricular macula or to the saccular macula (56, 99). In guinea pigs isolated loss of just the utricular macula in one labyrinth causes strong postural changes at the acute stage (yaw head turn, head roll-tilt toward the affected side) (99). These are similar to the responses found with complete unilateral vestibular loss since isolated unilateral utricular loss will upset the bilateral balance between the two VN just as a total unilateral loss does. These responses diminish over time in vestibular compensation. Comparable data from isolated utricular loss in human patients is rare (2). One patient inadvertently received what was probably an isolated utricular loss and was described as showing an “ocular tilt reaction” (61). The patient showed ipsilesional maintained torsional eye position, roll head tilt toward the affected ear and skew deviation with the ipsilesional eye being lower in the orbit.

In total unilateral vestibular loss, the saccular macula is destroyed as well as the utricular macula, but the interaction in the saccular system is predominantly between opposing sectors within each macula (cross-striolar inhibitory interaction within each macula) with very little commissural inhibitory interaction. Thus, the removal of one saccular macula in a unilateral labyrinthectomy should remove both opposing sectors and so not cause a bilateral imbalance of saccular activity. In guinea pigs, selective unilateral removal of just the saccular macula had little measurable effect on posture or oculomotor responses (56, 99). Such a result is consistent with cross-striolar inhibition because both interacting sectors within the one saccular macula are removed so there is no imbalance of saccular activity. There is a remaining saccular macula in the opposite labyrinth to signal GIF.

#### Galvanic Vestibular Stimulation (GVS)

**Figure 12:**
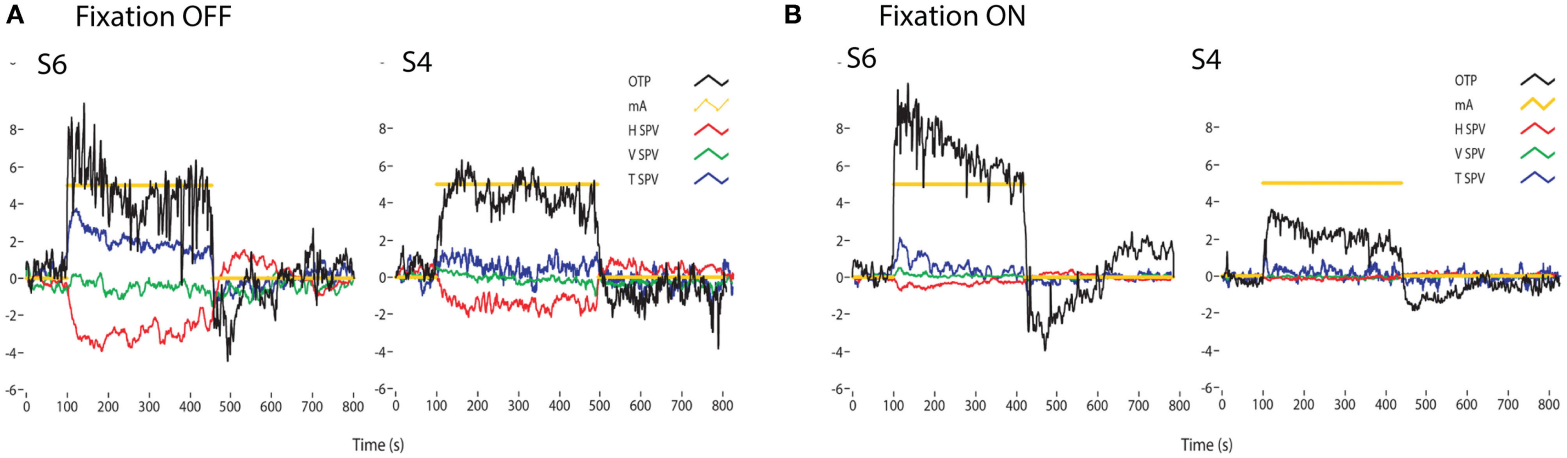
Time series of the eye movements of two healthy subjects (S1 and S2) to a square wave of bilateral galvanic stimulation with fixation off **(A)** and on **(B)**, showing the current (mA), the ocular torsion position (OTP) and horizontal (HSV), vertical (VSV) and torsional (TSV) eye velocity. The ordinate for each graph is degrees (for OTP) or degrees/sec for the eye velocity records. GVS causes large changes in torsion position, with and without fixation. In darkness the eye velocity responses are clear, confirming that GVS activates all semicircular canals as well as the otoliths. However, the importance of vision is shown by how the eye velocity responses are greatly reduced in the presence of a fixation point [from Figure 5 of (102)]. There is considerable variability between subjects for the same GVS stimulus. Reprinted by permission from Springer Nature, MacDougall et al. (102) © 2020.

All vestibular receptors and afferents from both canals and otoliths are activated by small cathodal and inhibited by small anodal currents (15), which in human subjects are usually passed between electrodes on the mastoids. This is bilateral Galvanic Vestibular Stimulation (GVS) and it usually consists of low current (5–10 mA or less) cathodal stimulation of one mastoid and simultaneous anodal stimulation of the opposite mastoid using surface electrodes with large surface areas [small area electrodes cause discomfort and even skin burns (100)]. With maintained (DC) stimulation this bilateral stimulus causes both eyes to adopt a rolled eye position, rolled away from the cathode. Since the response is a maintained torsional eye position rather than an eye velocity response it is held to be of otolithic origin. However, it is important to emphasize that GVS activates both canal and otolith receptors and afferents (15–17, 101). Neural recordings from all vestibular sensory regions show that GVS is not a specific otolithic stimulus—so caution is needed in interpreting the results of GVS as purely otolithic, although there is a clear otolithic component. The canal contribution becomes clear if the GVS is delivered in darkness where, in addition to the torsion, nystagmus is seen (Figure 12). Vision usually suppresses the GVS-induced nystagmus, as shown in Figure 12 it reduces the eye velocity response to GVS. Transient GVS stimulation has been proposed as a clinical test of peripheral function and it is affected in Meniere's Disease (103–105).

This mainly torsional eye movement response to GVS is in accord with the physiology discussed above. Cathodal galvanic stimulation of the left mastoid will activate all afferents from both sectors of the left utricular macula, and simultaneous anodal stimulation of the right mastoid will disfacilitate all afferents from both sectors of the right macula. As a result, afferents from the left medial sector will be facilitated, and those from the right medial sector will be disfacilitated, just as occurs during a real roll tilt of the head left ear down—toward the cathodal side. The result will be activation of afferents from the left utricular macula and simultaneous reduction of commissural inhibition from the right side since the afferents from the medial sector on the right have a reduced firing rate. This bilateral stimulus should cause maintained rolled ocular torsion opposite to the cathode because the stimulus pattern for the two medial sectors is comparable to that caused by a real roll-tilt to the left ear. The left lateral sector afferents will be activated, and the right lateral sector afferents will be silenced by the anodal current to the right mastoid. But just as discussed above for real roll-tilt, the contribution of the lateral sectors is apparently outweighed by the contribution from the medial sectors. It seems that during the GVS the activation of afferents from the lateral sectors (and their commissural interaction) should just cancel the effect of the stimulation of the medial sectors. One argument has been that this cancellation does not happen because the macula areas of the opposing receptors are not equal (9, 35). Another consideration is the very differential projections of the medial and lateral sectors shown by Maklad et al. (37). Afferents from the lateral sector project extensively to the cerebellum.

Unilateral galvanic stimulation causes smaller but clear eye movement responses. Unilateral cathodal stimulation of one mastoid will activate afferents from both sectors of the left utricular macula and so activate ipsilateral type I neurons from the medial sector and so cause both eyes to roll away from the cathodal side. Unilateral anodal stimulation of the right mastoid will disfacilitate the afferents from both sectors of the right utricular macula and so reduce the activity of the all afferents from the right including the right medial type I VN neuron. In turn that disfacilitation will reduce the inhibition acting on the left VN neuron, resulting in increased activation of the left VN neuron (via disinhibition) and so an ocular torsion response of both eyes. The enhanced activation should drive the response so the eyes should tort toward the anodal side. Both of these results have been reported (100, 102, 106).

For the saccular system left cathodal stimulation and right anodal stimulation will simultaneously facilitate afferents from dorsal and ventral sectors of the left saccular macula, and disfacilitate afferents from both sectors of the right saccular macula. Since there is little commissural interaction in the saccular system there should be little or no contribution from the saccular macula on the opposite side.

## Summary

Acutely after unilateral loss there are asymmetrical ocular responses to roll-tilt and to lateral linear acceleration. However, these asymmetries reduce over time such that long term patients (6–10 weeks) show no consistent asymmetry to roll-tilt stimuli (7). Similarly, patients 6 weeks after surgery show no asymmetry for linear lateral translations (81, 107). This is in sharp contrast to the semicircular canal system where response asymmetries after unilateral loss are permanent as shown by the head impulse test (108–110). Clinical tests based on asymmetry of oculomotor responses to roll-tilt or lateral translation do not show a reliable difference for the two opposite directions of gravitoinertial force so using these tests the clinician cannot determine whether the left or right utricular macula has been compromised. The clinical value of VEMPs is that they do allow identification of the affected side in unilateral loss and even allow the clinician to gauge whether it is the utricular or saccular macula (or both) which are affected in both acute and chronic patients. It seems that over time vestibular compensation takes place—and so except for the visual bias, unilateral centrifugation and VEMPs—the asymmetry between the two sides is reduced.

This review shows how the inhibitory interactions in the vestibular nuclei (VN) between neurons receiving afferents from the otolithic maculae [as summarized by (29, 33)] explain the results of several clinical and experimental tests of otolith function. Uchino has shown that inhibitory interaction in the vestibular nuclei (VN) is fundamental for the operation of the peripheral otolithic system (29). That within each macula there is inhibitory interaction across the striola (called cross-striolar inhibition) and in the case of the utricular macula there is additionally inhibitory interaction between the afferents from each labyrinth (commissural inhibitory interaction). The essential outcome of inhibitory interaction is that the one GIF stimulus will cause two sources of excitation of neurons in the vestibular nuclei (VN)—from both direct facilitation of some utricular receptors in one sector complemented by indirect excitation resulting from the disfacilitation from utricular receptors in the opposing sector. Utricular mutual commissural inhibitory interaction parallels the commissural inhibitory interaction between afferent input from the two horizontal semicircular canals to angular acceleration. It is important to emphasize the neurons showing this inhibitory interaction are only a small proportion of all the otolithic neurons—many otolithic neurons are outside the inhibitory interaction loops. There are effectively two complementary otolithic systems—the sustained system concerned with signaling low frequency GIF stimuli and the transient system which is activated by high frequency stimuli such as sounds and vibration (1). Most clinical tests of the sustained otolith system using low frequency GIF stimuli do not show unilateral loss reliably, whereas tests of transient otolith function do show unilateral otolithic loss. The transient otolithic system has been reviewed extensively recently (1, 21), so in this paper the focus has been on the sustained otolithic system.

## Author's Note

This review is dedicated to Bernard Cohen who made so many pioneering contributions to understanding vestibular function. It is a tribute to honor the great work that Yoshio Uchino carried out over so any years, illuminating the operation of the otoliths.

## Author Contributions

The author confirms being the sole contributor of this work and has approved it for publication.

## Conflict of Interest

The author declares that the research was conducted in the absence of any commercial or financial relationships that could be construed as a potential conflict of interest.

## Abbreviations

ACS: air conducted sound
BCV: bone-conducted vibration
OM: otolithic membrane
GIF: gravitoinertial force—the effective stimulus for otolithic receptors
HSV: horizontal slow phase eye velocity
VSV: vertical slow phase eye velocity
TSV: torsional slow phase eye velocity
SCD: semicircular canal dehiscence
LPR: line of polarity reversal
type I: excitatory neurons in the vestibular nucleus
type II: inhibitory neurons in the vestibular nucleus
VN: vestibular nucleus
ABD: abducens nucleus
GVS: galvanic vestibular stimulation
VEMP: vestibular evoked myogenic potential
oVEMP: ocular vestibular evoked myogenic potential
cVEMP: cervical vestibular evoked myogenic potential
Fz: the midline of the forehead at the hair line
OCR: ocular counterrolling—torsion of the eye in a direction opposite to the gravitoinertial force
OTR: the ocular tilt reaction. A triad of ipsilesional roll head tilt ipsilesional ocular torsion and skew deviation with the ipsilesional eye lower in the orbit
ipsilesional: on the same side as the loss or lesion
contralesional: on the opposite side to the loss or lesion (in other words the healthy side).
